# Diminished Renal Function and the Incidence of Heart Failure

**DOI:** 10.2174/157340309788970388

**Published:** 2009-08

**Authors:** Johan Ärnlöv

**Affiliations:** Department of Public Health and Caring Sciences, Uppsala University, Uppsala, and the Department of Health and Social Sciences, Högskolan Dalarna, Falun, Sweden

**Keywords:** Heart failure, kidney disease, glomerular filtration rate, creatinine, cystatin C, albuminuria.

## Abstract

Heart failure is one of the most common, costly, disabling and deadly diseases. During the last decade, several different indices reflecting renal function such as creatinine-based glomerular filtration rate, circulating levels of cystatin C and low-grade albuminuria have been demonstrated to be independent risk factors for heart failure. This review summarizes our current knowledge of the relationship between diminished renal function and the incidence of heart failure in the community, and also in individuals with increased risk of heart failure such as patients with overt cardiovascular disease, hypertension or diabetes. This review will also put forward important areas of future research in this field.

## INTRODUCTION

**Fig. (1):**
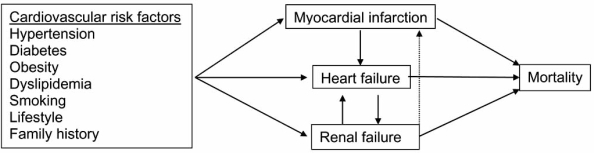
The interplay between the development of myocardial infarction, heart failure and renal failure.

There is growing recognition of the clinical importance of the symbiotic relationship between the kidney and the heart (Fig. **1**) [1]. It is well known that patients with heart failure often also develop renal failure, and renal impairment in heart failure patients has become increasingly recognized as an independent risk factor for morbidity and mortality [2-9]. A recent meta-analyses by Smith and co-workers [10] showed that a large proportion of heart failure patients had renal impairment of some degree, and that these patients had a 50% increased relative risk for mortality compared to heart failure patients with normal renal function. Moreover, almost a third of heart failure patients had moderate to severe renal impairment, with a more than 2-fold increased relative mortality risk and greater than 50% mortality during five years of follow-up. 

The temporal relationship between heart failure and renal failure appears to be complex, however, and the opposite chain of events is also common: patients with chronic kidney disease are at substantially higher risk of developing cardiovascular disease and heart failure. In fact, heart failure is about 15 times more common in patients with chronic kidney disease than in patients with normal renal function [11] and individuals with chronic kidney disease are more likely to die of cardiovascular disease than to develop end-stage renal disease [12, 13].

Although the close relationship between cardiovascular and renal pathologies is well recognized in advanced nephropathy and heart failure, it has received less attention in early disease. To date, several studies have suggested that diminished renal function may be involved in the development of heart failure at an early stage, prior to the development of clinically overt heart failure and kidney disease. The present review will focus on studies that have reported the longitudinal relationship between different indices of renal function (serum creatinine, creatinine-based equations of glomerular filtration rate, serum cystatin C, and albuminuria) and the development of heart failure in individuals without heart failure at baseline.

## SERUM CREATININE AND CREATININE-BASED EQUATIONS OF GLOMERULAR FILTRATION RATE AS RISK FACTORS FOR INCIDENCE OF HEART FAILURE

The serum levels of creatinine and creatinine-based equations of glomerular filtration rate have been used extensively in clinical practice, as well as in research, as indirect estimates of glomerular filtration rate. Although widely used, creatinine-based equations of glomerular filtration rate such as Cockroft-Gault [14] or the Modification of Diet in Renal Disease (MDRD) equation [15] have been shown to have limitations as estimates of glomerular filtration rate, particularly in the elderly and in individuals with glomerular filtration rate in the normal or near-normal range [16].

### Community-Based Studies

The association between elevated serum levels of creatinine and the incidence of heart failure in the community was first reported by Gottdiener and co-workers in the Cardiovascular Health Study [17]. In that investigation in an elderly study population, a comprehensive evaluation of different risk factors for heart failure was performed and serum levels of creatinine were suggested to contribute to 6% of the attributable risk of heart failure [17]. In a more recent report from the Cardiovascular Health Study, Fried and colleagues concluded that even in a sub-sample of healthy individuals and individuals with mild renal impairment (defined as serum creatinine >1.5 mg/dL), elevated serum creatinine was associated with an increased risk of heart failure [18]. The association between creatinine-based glomerular filtration rate and incidence of heart failure has since been validated in another cohort of elderly (the EPESE-study) [19] as well as in a cohort with a wider age range (the ARIC study)[20].

### High-Risk Populations

Serum creatinine levels as a predictor of heart failure has also been evaluated in individuals with high risk of heart failure such as patients with prevalent cardiovascular disease, hypertension, and/or diabetes. In most of these studies [21-23], however, heart failure has not been evaluated as the primary outcome. For instance, in secondary analyses of the Heart Outcomes and Prevention Evaluation (HOPE) study, mild renal insufficiency was associated with a doubled risk of heart failure in crude analyses but not after adjusting for other cardiovascular risk factors [21]. In the Valsartan in Acute Myocardial Infarction Trial (VALIANT) involving patients with an acute myocardial infarction[22], lower glomerular filtration rate (MDRD) was associated with a higher risk of heart failure in crude analyses. No multi-variable analyses were presented in that study. Moreover, a comparison of the predictive value of two different creatinine-based equations for glomerular filtration rate (Cockroft-Gault and MDRD) was performed in the Valsartan Antihypertensive Long-term Use Evaluation (VALUE) trial. Interestingly, in that study estimated glomerular filtration rate according to MDRD was a strong predictor of future heart failure events while estimated creatinine clearance from Cockroft-Gault was not [23].

In a report from the Heart and Estrogen/Progestin Replacement Study (HERS), the primary aim was to identify predictors of heart failure in post-menopausal women with coronary heart disease [24]. Bibbins-Domingo and co-workers reported that diabetes was the strongest risk factor for heart failure and that diabetic women with depressed creatinine clearance were those with the highest risk. The relevance of impaired renal function in individuals with diabetes is further supported by another study in men and women with diabetes, in which those with diabetic nephropathy were at increased risk of subsequent heart failure [25]. Importantly, in a post-hoc analysis of the Reduction of Endpoints in NIDDM with the Angiotensin II Antagonist Losartan (RENAAL) trial, patients with diabetes and more severe chronic kidney disease had higher incidence of heart failure, a trend that was reduced by treatment with losartan, an angiotensin receptor blocker [26].

## CYSTATIN C, A SUPERIOR RISK MARKER FOR INCIDENCE OF HEART FAILURE?

Circulating cystatin C concentration is a relatively novel measure of kidney function that has been suggested to overcome many of the limitations of using creatinine-based estimates, as the relationship between kidney function and cystatin C concentration does not appear to vary with age, sex, and body mass [16, 27, 28]. Recently, cystatin C has been suggested to be a more reliable indicator of kidney function than creatinine-based glomerular filtration rate, particularly in the elderly and in individuals with glomerular filtration rate within the normal range [16, 29, 30]. It should, however, be noted that the superiority of cystatin C is not undisputed, and it is yet to be fully established as the marker of choice for glomerular filtration rate in clinical practice [31].

### Community-Based Studies

The association between serum cystatin C and development of heart failure in the community was first reported by Sarnak and co-workers in the Cardiovascular Health Study [32]. Serum cystatin C concentrations were associated with a linear increase in risk across quintiles of cystatin C, in contrast to serum creatinine concentration or estimated GFR where there was a J-shaped association with heart failure. This study suggested that serum cystatin C concentrations provide a better measure of risk assessment than serum creatinine concentrations, particularly in the “normal” range of kidney function. This was further supported by a subsequent report from the Cardiovascular Health Study, where Shlipak and co-workers showed that higher serum concentrations of cystatin C independently predicted heart failure also in individuals without chronic kidney disease while creatinine-based glomerular filtration rate equations (MDRD) did not [33]. 

In secondary analyses in the Cardiovascular Health study, slightly stronger associations between cystatin C levels and heart failure were seen in blacks as compared to whites [32]. The question of ethnic differences in the association between renal impairment and the development of heart failure was investigated in greater depth by Bibbins-Domingo in the Health, Aging, and Body Composition Study [34]. In that study, the association between renal function and heart failure risk was stronger in older black individuals than in older white individuals, and this difference was particularly evident when cystatin C concentration was used to assess renal function. In fact, the population attributable risk of heart failure was 47% for blacks with moderate or high concentrations of cystatin C but only 5% in whites [34].

In a recent report from the Physicians’ Health study, Djousse and co-workers demonstrated that higher levels of cystatin C were associated with an increased risk of heart failure and that this association was evident in hypertensive individuals only [35]. This result differs from other community-based samples, where no interaction regarding hypertensive status has been reported.

One major limitation of most studies investigating the relationship between renal impairment and incidence of heart failure has been the lack of differentiation between systolic and diastolic heart failure. This important issue was recently addressed by Moran and co-workers in another report from the Cardiovascular Health Study [36] In that study, cystatin C levels were linearly associated with the incidence of systolic heart failure, whereas only the highest concentrations of cystatin C were predictive of diastolic heart failure [36], suggesting that there may be somewhat different pathophysiological pathways leading to these two types of heart failure.

### High-Risk Populations

Several previous studies have indicated that elevated cystatin C levels are predictive of cardiovascular morbidity and mortality in patients with prevalent coronary heart disease [37, 38], yet the association between cystatin C levels and the risk of heart failure in high-risk populations has been less studied. In a report from the Heart and Soul Study involving ambulatory patients with coronary heart disease, higher cystatin C levels were found to predict incident heart failure [39]. Interestingly, high cystatin C levels had similar predictive value for adverse clinical outcomes in individuals with or without microalbuminuria, suggesting that the association between higher cystatin C concentrations and risk of heart failure is not solely mediated by microalbuminuria. 

## LOW-GRADE ALBUMINURIA AND THE INCIDENCE OF HEART FAILURE

Although low-grade albuminuria has traditionally been considered to be an indicator of early renal pathology, there is an emerging view that albuminuria is also a marker of generalized vascular pathology such as endothelial dysfunction (the STENO hypothesis)[40]. Many prospective studies have suggested that increased risk of cardiovascular events begins well below the current threshold of microalbuminuria [41, 42], yet the association with heart failure has been less studied.

### Community-Based Studies

There have been a few community-based studies investigating the relationship between albuminuria and incidence of heart failure. In a study by Kistorp and co-workers [43], an increased urinary albumin/creatinine ratio was not found to be predictive of incidence of heart failure in a Danish cohort. It should be noted that in that study, incidence of heart failure was considered a secondary endpoint, and there was only a low number of heart failure events during follow-up (n = 19). 

In contrast, Ingelsson et al. reported that the urinary albumin excretion rate predicted heart failure incidence independently of both established risk factors for heart failure and other indices of renal function (creatinine-based glomerular filtration rate (MDRD) and serum cystatin C) in a community-based sample of elderly men (the Uppsala Longitudinal Study of Adult Men, ULSAM)[44]. Furthermore, urinary albumin excretion rate also remained an independent predictor of heart failure incidence in a sub-sample without myocardial infarction (either at baseline or during follow-up), suggesting that the rate of excretion of urinary albumin also predicts the incidence of non-ischemic heart failure.

### High-Risk Populations

Several authors have reported an association between albuminuria and heart failure in patients with diabetes. For instance, in the Appropriate Blood Pressure Control in Diabetes (ABCD) trial, patients with type 2 diabetes and macroalbuminuria were found to have a greater than threefold risk of heart failure as compared to diabetics with normo- or microalbuminuria [45]. Moreover, in the DIABHYCAR study (type 2 DIABetes, Hypertension, CArdiovascular Events and Ramipril), every 10-fold increase in urinary albumin concentration was associated with a more than doubled risk [46]. In two reports from the HOPE study, in patients either with diabetes or without diabetes, microalbuminuria [47] or any degree of albuminuria [42] was found to be predictive of heart failure, independently of established cardiovascular risk factors (including serum creatinine)[42]. 

The predictive importance of low-grade albuminuria in hypertensive individuals with left ventricular hypertrophy was shown in the LIFE study [48], where a urinary albumin/ creatinine ratio of > 3.5 mg/mmol was associated with a twofold higher risk of future heart failure. In that study, the participants with both albuminuria and ECG strain were at particularly high risk.

It is important to note that although there are convincing data to show that low-grade albuminuria is an independent predictor of heart failure, this association should not necessarily be construed to imply that there is a direct benefit in lowering of the albuminuria level. This essential issue was addressed in the landmark study by deZeeuw and co-workers in a report from the RENAAL trial. In that study, low-grade albuminuria was found to be an independent predictor of future heart failure in individuals with diabetic nephropathy, but more importantly, a 50% reduction in albuminuria after 6 months of antihypertensive treatment was associated with a 27% reduction in heart failure risk, supporting the idea of lowering of albuminuria as a treatment goal in order to achieve cardiovascular protection [49].

## THE CONJOINT EFFECT OF GLOMERULAR FILTRATION RATE AND ALBUMINURIA FOR THE PREDICTION OF HEART FAILURE

Even though several previous studies have shown that indices of glomerular filtration rate and albuminuria predict heart failure independently of each other, the conjoint effect of glomerular filtration rate and albuminuria for the prediction of heart failure is less reported. In a secondary analysis in the Cholesterol And Recurrent Events (CARE) trial [50], myocardial infarction patients with both proteinuria and impaired glomerular filtration rate (< 60 mL/min/1.73 m2) were more than twice as likely to develop heart failure as patients with one abnormality, or with neither. 

## FUTURE RESEARCH

Despite the relatively large number of studies, several important issues regarding the prognostic value of the indices of renal dysfunction and the development of heart failure remain unresolved. First, there have been no studies evaluating whether adding the indices of diminished renal function to a model with established risk factors for heart failure will improve the model discrimination, calibration, and global model fit for the prediction of heart failure [51-54]. It is essential that this issue be addressed in order for these indices to be used in clinical practice, to identify individuals who have an elevated risk of heart failure. Second, no ideal cut-offs for the indices of renal impairment have been identified. The thresholds used have either been based on classification of renal disease or on the distribution of the variable of interest in a particular sample. Future studies must identify relevant thresholds for the different renal indices in order to achieve optimal model discrimination. Thirdly, whether or not the conjoint effect of glomerular filtration rate and albuminuria conveys additional prognostic information has not been well studied. Fourthly, it is important to note that heart failure was not the primary outcome in most studies in high-risk populations. Fifth, most community-based studies were mainly performed in samples taken from the elderly and our knowledge of the predictive capacity of the indices of renal function is limited in individuals who are less than 65 years old. Sixth, patients with renal dysfunction are often excluded from intervention trials and our knowledge of whether intervention is beneficial in this patient group is limited. There is a need for more studies that evaluate the benefit of improvement of renal function in lowering the risk of heart failure. Finally, there have been few data on the prediction of different subtypes of heart failure such as diastolic heart failure and non-ischemic heart failure.

## CONCLUSIONS

There is a considerable body of data to support the notion that diminished renal function, as evaluated by reduced glomerular filtration rate or low-grade albuminuria, is an independent risk factor for subsequent heart failure both in high risk populations such as patients with overt cardiovascular disease, hypertension or diabetes, and in the community. Cystatin C appears to be a superior marker for future risk of heart failure compared to serum levels of creatinine or creatinine-based equations of glomerular filtration rate. Moreover, the risk of heart failure appears to begin at levels of glomerular filtration rate and albuminuria that are considered to be in the normal range according to current clinical practice. Some data suggest that the relative importance of renal dysfunction for the development of heart failure is different in different ethnic groups, an issue that merits further studies. Thus, based on the studies presented in this review, the close association between renal and cardiac dysfunction already appears to be present before the development of clinically overt renal and cardiovascular disease, an observation that challenges the current definition of cardiorenal syndrome [1].
